# Evaluation of Infections Caused by Carbapenem-Resistant *Pseudomonas aeruginosa*, *Acinetobacter baumannii*, and *Klebsiella pneumoniae* in an Intensive Care Unit: A Retrospective Study

**DOI:** 10.3390/antibiotics14070700

**Published:** 2025-07-12

**Authors:** Elif Kerimoglu, Tuba Catak, Anil Kilinc

**Affiliations:** 1 Department of Intensive Care, Ordu Training and Research Hospital, Ordu 52200, Türkiye; 2 Department of Anesthesiology and Reanimation, Faculty of Dentistry, Necmettin Erbakan University, Konya 42090, Türkiye; tuba.catak@erbakan.edu.tr; 3 Department of Anesthesiology and Reanimation, Faculty of Medicine, Ordu University, Ordu 52200, Türkiye; anilkilinc@odu.edu.tr

**Keywords:** carbapenem, carbapenem resistance, intensive care, Gram-negative bacteria, mortality, risk factors, platelet count, antibiotic therapy

## Abstract

**Objectives:** Carbapenem-resistant Gram-negative bacteria (CR-GNB) infections in intensive care units (ICUs) are increasingly prevalent and associated with high mortality. This study aimed to investigate the distribution of isolated bacteria and determine the factors associated with mortality among ICU patients diagnosed with CR-GNB infections. **Methods:** This retrospective study included 95 patients admitted to the ICU between February 2022 and July 2024 who were diagnosed with CR-GNB infections via culture and initiated on treatment. Thirty-day mortality was defined as the clinical outcome, and patients were divided into two groups: survivors (Group 1, *n* = 42) and deceased (Group 2, *n* = 53). Demographic, clinical, laboratory, and microbiological data were analyzed. **Results:** Advanced age, the presence of malignancy, an elevated Charlson Comorbidity Index (CCI), lower platelet counts, and higher C-reactive protein (CRP) levels were significantly associated with mortality (*p* < 0.05). Trauma-related admissions were more common among survivors, while sepsis-related admissions predominated among non-survivors. No statistically significant associations were observed between antibiotic regimen type and mortality. Culture-based pathogen distribution revealed *A. baumannii* as the predominant organism in respiratory samples, while *K. pneumoniae* was more frequently isolated from bloodstream and urinary specimens. **Conclusions:** Mortality in ICU patients with CR-GNB infections is influenced by both baseline comorbidities and infection-related inflammatory markers. This study provides region-specific insights from a high-resistance ICU setting and may inform risk stratification, prognostication, and management strategies in critically ill patients with CR-GNB infections.

## 1. Introduction

Intensive care units (ICUs) are among the hospital settings with the highest burden of antibiotic-resistant pathogens. In particular, carbapenem-resistant *Enterobacterales* (CRE), *Acinetobacter baumannii* (CRAB), and *Klebsiella pneumoniae* (CRKP) cause difficult-to-treat infections in healthcare settings and are associated with high mortality rates [1,2]. These pathogens are often resistant to penicillins, cephalosporins, carbapenems, monobactams, and β-lactamase inhibitor combinations [3]. Treatment options are extremely limited—many isolates remain susceptible only to “last-resort” antibiotics such as colistin or tigecycline, and some have developed resistance even to these agents [4,5]. Recently, the efficacy of new antibiotics against carbapenem-resistant Gram-negative bacteria (CR-GNB), such as ceftazidime–avibactam, ceftolozane–tazobactam, meropenem–vaborbactam, imipenem–cilastatin–relebactam, plazomicin, eravacycline, and cefiderocol, has been investigated, and some of these antibiotics have entered clinical use [6]. Despite these developments, the World Health Organization has ranked carbapenem-resistant *Enterobacteriaceae* and *A. baumannii* among the highest priority pathogens for new antibiotic development [7].

Epidemiological data show that the burden of CR-GNB is rising in many regions. In Europe, the incidence of CRKP bloodstream infections reached approximately 3.97 per 100,000 population in 2023—a 57.5% increase compared to 2019 [8]. This upward trend counteracts public health targets and has been observed in 23 EU countries, indicating widespread hospital transmission of these resistant strains [8]. CRAB is also alarmingly high; some southern and eastern European countries report that >90% of *Acinetobacter* isolates are non-susceptible to carbapenems [9]. Beyond Europe, regions such as the Middle East and Latin America likewise face very high rates of multidrug-resistant *Acinetobacter baumannii* including CRAB strains often exceeding 70% of isolates [10]. The widespread use of broad-spectrum antibiotics has fostered new resistance mechanisms [11,12]. These trends illustrate that carbapenem-resistant infections are a growing problem worldwide, affecting both developed and developing healthcare systems. Moreover, CR-GNB infections are common in the ICU and present a serious danger to critically ill patients in regional and international areas [13,14]. Even data from local settings, such as our ICU, contribute to the understanding of this worldwide challenge and may inform broader infection control and antimicrobial stewardship efforts. Therefore, identifying prognostic risk factors in patients exposed to CR-GNB in ICU settings is of critical importance.

The predominant mechanism of resistance in CRE is the production of carbapenem-hydrolyzing β-lactamases (carbapenemases) [15]. According to the Ambler classification, carbapenemases fall into three main classes—A, B, and D—that have evolved in *Enterobacterales* [16]. Class A comprises serine-based enzymes such as *Klebsiella pneumoniae* carbapenemases (KPCs), which have spread globally and become endemic in certain areas. Class B includes the metallo-β-lactamases (MBLs)—for example, New Delhi MBL (NDM), Verona integron-encoded MBL (VIM), and IMP-type enzymes—with NDM-1 being one of the most widely disseminated worldwide. Class D carbapenemases are represented by oxacillinase enzymes like OXA-48, a serine enzyme that efficiently hydrolyzes carbapenems [17]. Notably, oxacillinase-48 (OXA-48)-like carbapenemases were first identified in Turkey and are now prevalent across neighboring regions [18]. By contrast, KPC enzymes (Ambler class A) were initially reported in the United States but have since become endemic in parts of Europe (e.g., Greece and Italy) and elsewhere [3]. Together, KPC, NDM, VIM, IMP, and OXA-48 variants are often referred to as the “big five” carbapenemases due to their global dominance in CRE [19]. The spread of these resistance genes is facilitated by plasmids and international patient transfers, enabling carbapenem-resistant strains to emerge in diverse healthcare settings [3]. This scenario poses a serious challenge for infection control and highlights the importance of the surveillance and characterization of local resistance patterns.

Despite the clear threat posed by infections from multidrug-resistant Gram-negative bacteria (MDR-GNB), there is a paucity of data on the factors that predict outcomes for infected ICU patients. In many high-burden settings, surveillance and clinical data on MDR-GNB infections remain limited [20,21], making it difficult to identify which patient or infection characteristics drive mortality risk. Therefore, this study aimed to investigate the distribution of isolated bacteria and determine the factors associated with mortality among ICU patients diagnosed with CR-GNB infections.

## 2. Results

A total of 95 patients were included in the study. The median age was 66.7 ± 21.8 years in survivors and 76.6 ± 10.5 years in deceased patients. Among female patients, 20 (43.5%) survived, while 26 (56.5%) were deceased. Among male patients, 22 (44.9%) survived, and 27 (55.1%) were deceased. The mean length of pre-ICU hospitalization was 1 day in both groups. The median length of ICU stays before pathogen isolation was 11 days in both survivors and deceased patients. There was no statistically significant difference between the two groups in terms of age, gender, length of pre-ICU hospitalization, and length of ICU stay before pathogen isolation (*p* > 0.05).

The distribution of comorbid conditions did not differ between deceased and surviving patients. However, the frequencies of malignancy, cardiovascular disease, and chronic respiratory disease were modestly elevated in deceased patients compared to surviving patients. Furthermore, the overall comorbidity burden, as measured by the CCI, was greater among deceased patients compared to surviving patients (five vs. four, *p* = 0.024). Accordingly, the proportion of patients with moderate (45% vs. 36%, *p* = 0.004) and severe CCI scores was higher in the deceased group than in the survivor group (45% vs. 36% for moderate CCI; 55% vs. 45% for severe CCI; *p* = 0.004).

Among patients admitted to the ICU with sepsis, 3 (21.4%) survived, while 11 (78.6%) were deceased. Septic shock as the reason for admission was significantly more frequent in the deceased group. Among patients admitted to the ICU due to trauma, 11 (68.8%) survived, while 5 (31.2%) were deceased. Trauma-related admissions were statistically significantly more frequent among the survivors. No significant differences in mortality rates were observed for patients admitted due to respiratory failure or cardiovascular disease. Overall, a statistically significant association was found between the reason for ICU admission and mortality (*p* < 0.05). However, no significant associations were observed between mortality and infection type, mechanical ventilation, central venous catheterization, urinary catheterization, percutaneous endoscopic gastrostomy (PEG) tube presence, isolated Gram-negative microorganisms, or antibiotic regimen (*p* > 0.05). The median duration of treatment was 14 days in survivors and 6 days in deceased patients. Treatment duration was statistically significantly longer in the survivor group compared to the deceased group (*p* < 0.05).

The mean white blood cell (WBC) count was 12,774.52 ± 7857.13 cells/mm^3^ in the survivor group and 14,117.19 ± 7277.05 cells/mm^3^ in the deceased group. The mean leukocyte count was 10,481.21 ± 7295.85 cells/mm^3^ in the survivor group and 12,316.96 ± 6822.24 cells/mm^3^ in the deceased group. There was no statistically significant association between mortality and WBC or leukocyte counts (*p* > 0.05).

The mean platelet count was 252,619.05 ± 111,904.57 cells/mm^3^ in the survivor group and 172,450.94 ± 110,901.36 cells/mm^3^ in the deceased group; this difference was statistically significant (*p* < 0.05). CRP levels were 123.29 ± 86.65 mg/dL in the survivor group and 166.98 ± 101.46 mg/L in the deceased group. CRP levels were significantly higher in the deceased group (*p* < 0.05). No statistically significant associations were found between mortality and steroid use within the last 30 days, prior antibiotic history, diabetes mellitus, or surgical history (*p* > 0.05) (Table 1).

Significant risk factors associated with mortality included advanced age, type of disease, low platelet levels, and elevated CRP levels (*p* < 0.05). Each 1-year increase in age raised the mortality risk by 1.020-fold (*p* < 0.05). Patients with malignancy had a 2.23-fold higher risk of mortality compared to those without (*p* < 0.05). A one-point increase in the CCI was associated with a 1.22-fold higher risk of mortality (*p* < 0.05). A one-unit decrease in platelet count increased mortality risk by 1.002-fold (1/HR = 0.998), whereas a one-unit increase in CRP level raised mortality risk by 1.003-fold (*p* < 0.05) (Table 2).

The most frequently used treatment regimen was combination antibiotic therapy (*n* = 40), while beta-lactam/beta-lactamase inhibitor combinations were the least preferred (*n* = 24). No significant association was observed between antibiotic regimens and mortality or survival outcomes (*p* > 0.05).

The most frequently isolated microorganism in urine cultures was *K. pneumoniae* (*n* = 11, 27%). In tracheal aspirate cultures, *A. baumannii* (*n* = 28, 61%) was the predominant pathogen. *K. pneumoniae* (*n* = 11, 27%) was the most common pathogen in bloodstream and catheter-related infections. In wound infections, *A. baumannii* and *K. pneumoniae* were isolated at equal rates (*n* = 3 each, 7%). *P. aeruginosa* was the least common microorganism across all infection types. No statistically significant differences in mortality were observed based on microbial distribution (*p* > 0.05) (Table 3).

## 3. Discussion

Advanced age has long been recognized as a major determinant of adverse outcomes in critically ill patients, particularly in those with sepsis and multidrug-resistant infections. Our findings are consistent with the previous literature showing that aging is associated with increased vulnerability to infection-related mortality. Martín et al. reported that older age is strongly correlated with poor sepsis outcomes, largely due to factors such as increased comorbidity burden, frailty, and age-related immune decline (immunosenescence) [22]. Immunosenescence not only impairs the initial immune response to infection but also contributes to dysregulated inflammation and progression to organ dysfunction, which may explain the disproportionately higher mortality among elderly ICU patients with CR-GNB infections.

While pre-ICU hospital length of stay is recognized as a potential risk factor for MDR-GNB and mortality [23,24,25], some studies have reported no association with mortality [26,27]. Variations observed across studies could be attributable to heterogeneity in patient selection criteria. However, our results likely reflect the unique patient flow in our institution: the majority of ICU admissions originate directly from the emergency department, meaning these critically ill patients have minimal ward exposure before transferring to intensive care. Such rapid transitions to the ICU can limit their contact with hospital flora prior to ICU admission, potentially reducing the colonization risk associated with prolonged hospitalization. As a result, in this particular cohort, the duration of prior hospital stay had less opportunity to influence MDR colonization status or subsequent mortality, which helps explain why neither pre-ICU stay length nor time-to-isolation was significantly linked to mortality in our study.

Our findings reinforce the established association between high-risk comorbidities—particularly malignancy—and adverse outcomes in ICU patients with MDR infections. Previous studies have shown that patients with malignancy are especially vulnerable to poor outcomes in the context of bloodstream infections caused by resistant organisms such as extended-spectrum β-lactamase-producing *Enterobacteriaceae* (ESBL-PE) [28]. In line with these findings, our study demonstrated that patients with malignancy had a notably elevated risk of mortality. Additionally, the cumulative comorbidity burden, quantified by the CCI, emerged as a significant predictor of mortality. This aligns with prior studies in which higher CCI scores were independently associated with increased short-term mortality in resistant infections, including ESBL-producing *E. coli* bacteremia [29] and carbapenem-resistant *Enterobacteriaceae* infections [30]. These observations underscore the importance of incorporating comorbidity-based risk stratification—beyond individual diagnoses—when managing critically ill patients with bloodstream infections in the ICU.

Our results indicated that sepsis was more frequently observed among non-survivors, whereas trauma-related admissions were more common in the survivor group. This pattern is consistent with the findings of Mann et al., who reported lower sepsis-related mortality in trauma patients, potentially due to differences in inflammatory response dynamics or host physiology [31]. In contrast, respiratory failure and cardiovascular disease did not show significant associations with mortality, which is in line with prior studies such as that of Tertemiz et al. in patients with chronic respiratory comorbidities [32].

In this study, treatment regimens were not significantly associated with mortality. In particular, using combination therapy did not show a clear survival advantage over monotherapy in our data. This result mirrors the findings of at least one large study of CRKP bloodstream infections, which reported an overall 30-day mortality of 33% with no significant outcome differences between monotherapy versus combination regimens (including dual therapy and/or the addition of a β-lactam) [33]. That study found that mortality was driven more by patient factors (like severity of illness, indicated by high Pitt bacteremia scores) than by the specific antibiotic regimen, once the isolates were phenotypically highly resistant [33]. It is important to note, however, that the question of monotherapy versus combination therapy in treating carbapenem-resistant infections is still a matter of debate, and outcomes can vary depending on the scenario. Some studies (often from the pre-ceftazidime–avibactam era) did suggest that combination therapy (using two or more active drugs) was associated with better survival in CRKP infections [34,35,36]. These combinations typically involved adding agents like colistin, tigecycline, or an aminoglycoside to a backbone regimen. Similarly, in CRKP bloodstream infections, treatment with ceftazidime–avibactam has been associated with significantly higher survival compared to conventional regimens [37,38]. On the other hand, a meta-analysis reported that ceftazidime–avibactam, whether administered alone or in combination with colistin or an aminoglycoside, did not provide any additional survival benefit [13]. Although no significant association was observed between antibiotic regimens and mortality, treatment duration was longer in survivors than in deceased patients. Longer courses of treatment probably reflect early pathogen detection and timely therapy start, which may link to better clinical results.

In our cohort, the most frequently isolated microorganism in urine cultures was *K. pneumoniae*, while *A. baumannii* was the most frequently isolated microorganism in tracheal aspirate cultures. *K. pneumoniae* was also the dominant pathogen in bloodstream and catheter-related infections. In wound infections, *A. baumannii* and *K. pneumoniae* were isolated at equal frequencies. *P. aeruginosa* was the least common pathogen across all infection sources. Nevertheless, neither the specific microbial species isolated nor the infection sources demonstrated a significant correlation with patient mortality. In contrast, a study by Chou et al. reported that the anatomical site of infection influenced mortality in septic patients, with abdominal, respiratory, and biliary tract infections associated with higher mortality rates [39]. Despite the highest isolation rates in tracheal aspirate cultures in the present study, no significant mortality differences were observed compared to blood, urine, or wound cultures.

In adult ICU patients with carbapenem-resistant Gram-negative infections (CRKP, CRAB, and carbapenem-resistant *P. aeruginosa*), invasive interventions have been variably associated with mortality. Many studies report that the need for invasive mechanical ventilation correlates with higher death rates in these infections [13,40]. A previous ICU study found that mechanical ventilation was an independent predictor of mortality in CRKP infections [41]. Similarly, in carbapenem-resistant P. aeruginosa bacteremia, patients requiring mechanical ventilation or other devices (e.g., central lines, urinary catheters, or chest tubes) had markedly higher fatality rates [42]. However, data concerning CRAB infections remain contradictory. While certain cohorts did identify mechanical ventilation as a significant risk factor for death [40], a 2019 meta-analysis found no overall association between ventilator use and mortality in CRAB cases [43]. Notably, that meta-analysis *did* find that some invasive devices—especially urinary catheters—were independent predictors of higher mortality (approximately tripling the odds of death) in CRAB infection [43]. In contrast, routine ICU interventions like central venous catheters or enteral feeding tubes (PEG/nasogastric) have not consistently emerged as independent risk factors, likely because their use is nearly universal or reflects underlying illness severity rather than a direct causal effect [42].

Decreased platelet counts at infection onset and elevated CRP levels were also associated with higher mortality. These findings are also supported by the limited studies conducted in high-resistance regions where MDR-GNB patients are treated. Thrombocytopenia has been linked to bleeding complications, prolonged ICU stays, and increased mortality in critically ill patients [44]. Moreover, Baddal et al. recently applied machine learning to 788 ICU patients with carbapenem-resistant Gram-negative infections and identified CRP and platelet–large cell ratio among the top predictors of mortality, underscoring the prognostic value of these biomarkers in this setting [45]. These biomarkers likely reflect the host’s inflammatory response and degree of physiological decompensation. CRP, as an acute-phase reactant, is elevated in uncontrolled infection, while reduced platelet counts may indicate disseminated intravascular coagulation, bone marrow suppression, or consumption due to systemic inflammation. Given the high mortality risk in patients with MDR-GNB infections, early identification of such laboratory abnormalities can assist clinicians in risk stratification and prognostic assessment. Regular monitoring of CRP and platelet dynamics—especially when combined with clinical severity scores—may improve timely therapeutic decision-making and facilitate targeted supportive care [46].

### Study Limitations

This study has several limitations. First, it was a single-center, retrospective analysis with a relatively small sample size, which may limit the generalizability of the findings to other populations and healthcare settings with different epidemiological dynamics. Second, the retrospective nature of the study precluded the ability to control for all potential confounders. Third, molecular epidemiological methods such as clonality analysis or carbapenemase gene typing (e.g., MLST, PFGE, and PCR) were not performed, due to the lack of stored bacterial isolates and the retrospective design. This prevented us from determining whether infections originated from clonal spread or were polyclonal in nature—information that is particularly valuable in ICU outbreak settings. Numerous recent investigations have demonstrated that clonally related strains—particularly in outbreaks of carbapenem-resistant A. baumannii, K. pneumoniae, and P. aeruginosa—may dominate in high-resistance settings, enabling rapid intra-hospital spread [47,48,49]. Moreover, the detection of high-risk clones (e.g., ST11, ST235, and ST2) has been shown to inform targeted infection control strategies and treatment choices in both outbreak and surveillance contexts [49,50,51]. Fourth, because of a hospital-wide transition to a new electronic health record (EHR) system in May 2024, archived antimicrobial susceptibility data from earlier cases could not be retrieved reliably. As a result, we were unable to present a comprehensive analysis of resistance beyond carbapenem susceptibility, such as resistance to aminoglycosides, fluoroquinolones, or newer agents. Furthermore, due to the retrospective design and limitations in microbiological methodology, hetero-resistance—a phenomenon increasingly recognized in CR-GNB infections—could not be assessed. Future multicenter, prospective studies with integrated molecular surveillance and longitudinal resistance profiling are warranted to further elucidate the epidemiology, clonal dynamics, and treatment outcomes of CR-GNB infections in critically ill populations.

## 4. Materials and Methods

The study was conducted at a tertiary university hospital with a 14-bed mixed ICU, admitting approximately 850 patients annually. Data from 95 patients admitted to the resuscitation ICU between February 2022 and July 2024, who were diagnosed with hospital-acquired infections caused by CR-GNB isolated from body fluid or tissue samples and initiated on antibiotic therapy, were evaluated in this retrospective study. The infections included bloodstream infections, catheter-related bloodstream infections, ventilator-associated pneumonia (VAP), urinary tract infections (UTIs), and wound site infections (WSIs). Data were retrospectively obtained by reviewing the hospital’s electronic health record system (EHRS) and patient files.

### 4.1. Ethical Considerations

Ethical approval for this study was granted by the Ordu University Non-Interventional Clinical Research Ethics Committee (Decision No.: 2024/204; dated 6 December 2024). Informed consent was obtained from all subjects involved in the study.

### 4.2. Inclusion and Exclusion Criteria

Patients aged ≥ 18 years who were transferred to the adult ICU after hospitalization in the same institution or another center (e.g., emergency department or operating room) were included.

For cases with multiple CR-GNB infections, only the first infection was included in the risk factor analysis. Exclusion criteria included patients aged < 18 years, those admitted to the ICU after elective surgery, ICU stay < 24 h, and patients with polymicrobial culture growth.

Data were collected from the hospital’s EHRS and patient files. Variables included age, sex, comorbidities (cardiovascular/respiratory diseases, diabetes mellitus, and malignancies), laboratory findings recorded on the day the culture result confirmed CR-GNB (C-reactive protein [CRP], neutrophil, leukocyte, and platelet counts), history of surgical procedures within the last 30 days, antibiotic use (carbapenem, beta-lactam/beta-lactamase inhibitor, combination therapy, quinolone, cephalosporin, or none), corticosteroid use, the reason for ICU admission (sepsis, respiratory failure, trauma, and cardiac arrest), length of hospital stay prior to ICU transfer, and length of ICU stay until CR-GNB isolation. Additionally, invasive procedures at infection onset (urinary catheter, central venous catheter, mechanical ventilation, and percutaneous gastrostomy tube), isolated microorganisms (*P. aeruginosa*, *A. baumannii*, and *K. pneumoniae*), and specimen types (urine, tracheal aspirate, blood/catheter, and wound) were recorded. The type of antibiotic used in treatment, the duration of treatment, and 30-day mortality were assessed. Clinical outcome was defined as 30-day mortality, with survivors classified as Group 1 and deceased patients as Group 2.

### 4.3. Definitions

The comorbidity burden was calculated using the scoring system previously defined by Charlson et al., known as the Charlson Comorbidity Index (CCI) [52]. Cardiovascular disease (CVD) was defined as a history of cardiac arrhythmia, ischemic heart disease, chronic heart failure, peripheral vascular disease, or cerebrovascular disease. Patients were stratified into three categories based on their CCI scores: mild (1–2), moderate (3–4), and severe (≥5) [53].

Infection types, sepsis, and antibiotic resistance were defined according to international guidelines. Bloodstream infections, catheter-related bloodstream infections, VAP, UTIs, and WSIs were evaluated using the National Healthcare Safety Network surveillance criteria by the U.S. Centers for Disease Control and Prevention [54]. Sepsis was diagnosed per the Surviving Sepsis Campaign 2021 guidelines, requiring confirmed infection and a ≥2-point increase in the Sequential Organ Failure Assessment score to indicate organ dysfunction [55].

Carbapenem resistance was defined as the in vitro resistance of Gram-negative bacteria (e.g., *K. pneumoniae* and *A. baumannii*) to imipenem, meropenem, or ertapenem, confirmed via culture. Resistance levels were interpreted using antibiogram results and European Committee on Antimicrobial Susceptibility Testing clinical breakpoints [56].

Antimicrobial treatment was guided by institutional protocols and infectious disease consultation. Beta-lactam/β-lactamase inhibitor therapy involved ceftazidime–avibactam, and combined antibiotic therapy was described as using a carbapenem together with amikacin.

### 4.4. Statistical Analysis

Data were analyzed using IBM SPSS Statistics 26.0 (IBM Corp., Armonk, NY, USA) statistical analysis software. Descriptive statistics were reported as frequency (*n*), percentage (%), mean (X), standard deviation (SD), median (M), and minimum (min)–maximum (max) values.

The normality of continuous variables was assessed using the Shapiro–Wilk test. Non-normally distributed variables were compared with the Mann–Whitney U test, while categorical variables were analyzed using Pearson’s chi-square test or Fisher’s exact test.

Cox regression analysis was used to evaluate the impact of variables on survival. In addition, mortality-related factors were analyzed using life table analyses. A *p*-value of <0.05 was considered statistically significant in all analyses.

## 5. Conclusions

Infections caused by CR-GNB continue to pose a major clinical challenge, particularly in ICU settings. This study highlights outcome-associated factors in patients with MDR infections within a real-world tertiary ICU setting in Türkiye, providing crucial, region-specific insights into the distribution of resistant pathogens and associated risk factors. Our study identified several factors significantly associated with mortality in ICU patients diagnosed with CR-GNB infections, including advanced age, the presence of comorbidities—especially malignancy—low platelet counts, and elevated C-reactive protein (CRP) levels. Additionally, the CCI was higher among non-survivors, underscoring the prognostic value of the cumulative comorbidity burden. This study contributes region-specific data from a high-resistance setting and highlights the distribution patterns of CR-GNB by specimen type. Such local data substantially enhance the global understanding of antimicrobial resistance and help guide effective infection control and antimicrobial stewardship strategies.

## Figures and Tables

**Table 1:** Comparison of demographic and hospitalization characteristics according to mortality status.

	Group 1 (Survivor) *n = 42*	Group 2 (Deceased) *n = 53*	*p*
Age, year	66.7 ± 21.8	76.6 ± 10.5	0.078
Gender, female/male (*n*, %)	20 (48)/22 (52)	26 (49)/27 (51)	0.889
Comorbidity (*n*, %)			
Diabetes mellitus	15 (36)	13 (25)	0.264
Malignancy	4 (9)	13 (24)	0.066
Cardiovascular disease	10 (24)	19 (36)	0.264
Neurological disease	11 (26)	14 (26)	0.999
Chronic pulmonary disease	7 (17)	22 (23)	0.607
Chronic kidney disease	1 (2)	2 (4)	0.999
Charlson Comorbidity Index	4 (1–6)	5 (3–8)	0.024
Mild (*n*, %)	8 (19)	0	
Moderate (*n*, %)	15 (36)	24 (45)	0.004
Severe (*n*, %)	19 (45)	29 (55)	
Reason for intensive care admission (*n*, %)			0.041
Sepsis, septic shock	3 (7)	11 (21)	
Respiratory failure	23 (55)	34 (64)	
Trauma	11 (26)	5 (9)	
Cardiovascular arrest	5 (12)	3 (6)	
Length of pre-intensive care hospitalization, days	1 (1–40)	1 (1–30)	0.664
Length of intensive care stay before CR-GNB isolation, days	11 (3–40)	11 (1–65)	0.652
Type of infection (*n*, %)			0.326
Urinary tract infection	10 (24)	7 (13)	
Ventilator-associated pneumonia	18 (43)	29 (55)	
Bloodstream and catheter-related bloodstream infection	9 (21)	14 (26)	
Wound site infection	5 (12)	3 (6)	
Mechanical ventilator (*n*, %)	34 (81)	39 (74)	0.398
Central venous catheterization (*n*, %)	23 (55)	32 (60)	0.582
Urinary catheterization (*n*, %)	38 (90)	46 (87)	0.577
Percutaneous endoscopic gastrostomy tube (*n*, %)	12 (29)	8 (15)	0.110
Isolated Gram-negative bacteria (*n*, %)			0.243
*Pseudomonas aeruginosa*	2 (5)	6 (11)	
*Acinetobacter baumanii*	24 (57)	22 (42)	
*Klebsiella pnemonia*	16 (38)	25 (47)	
Antibiotics administered in treatment (*n*, %)			0.227
Carbapenem	15 (36)	16 (30)	
Beta-lactam/beta-lactamase inhibitor	7 (17)	17 (32)	
Combination therapy	20 (48)	20 (38)	
Duration of treatment, days	14 (5–26)	6 (1–14)	<0.001
WBC, cells/mm^3^	11,530 (2320–42,000)	13,710 (680–33,370)	0.173
Leukocytes, cells/mm^3^	9095 (1800–38,000)	12,110 (590–32,401)	0.085
Platelets, cells/mm^3^	223,000 (54,000–552,000)	141,000 (12,000–415,000)	0.001
CRP, mg/dL	107.5 (7–335)	149 (9–468)	0.037
Steroid use in the last 30 days (*n*, %)	27 (64)	26 (49)	0.138
Antibiotic use in the last 30 days (*n*, %)			0.461
Combination therapy	22 (52)	22 (42)	
Carbapenem	1 (2)	6 (11)	
Quinolone	3 (7)	4 (8)	
Beta-lactam/beta-lactamase inhibitor	11 (26)	11 (21)	
Cephalosporin	3 (7)	4 (8)	
No history of antibiotics	2 (5)	6 (11)	
Surgery in the last 30 days (*n*, %)	21 (50)	16 (30)	0.079

Abbreviations: Mann–Whitney *U* test (*Z*); chi-square test (*χ*^2^). Descriptive statistics are presented as mean (X), standard deviation (SD), median (M), minimum (min), maximum (max), number (*n*), and percentage (%).

**Table 2:** Results of Cox regression analysis of clinical variables and laboratory analysis.

	Exp (*β*) (%95 CI)	*p*	Model Significance	
			*χ* ^2^	*p*
Age	1.020 (1.002–1.038)	0.032	4.690	0.030
Gender (ref: female)	0.942 (0.550–1.615)	0.828	0.047	0.828
Comorbidity (*n*, %)				
Diabetes mellitus	0.651 (0.334–1.272)	0.209	1.602	0.206
Malignancy	2.232 (1.180–4.220)	0.014	6.431	0.011
Cardiovascular disease	1.210 (0.685–2.136)	0.511	0.432	0.510
Neurological disease	0.836 (0.445–1.572)	0.579	0.309	0.578
Chronic pulmonary disease	1.116 (0.583–2.137)	0.739	0.111	0.739
Chronic kidney disease	1.171 (0.284–4.833)	0.827	0.048	0.824
Charlson Comorbidity Index	1.217 (1.028–1.440)	0.023	5.183	0.023
Reason for intensive care admission (ref: sepsis)			5.638	0.131
Respiratory failure	0.762 (0.386–1.505)	0.434		
Trauma	0.330 (0.114–1.052)	0.060		
Cardiovascular arrest	0.405 (0.113–1.453)	0.166		
Duration of pre-intensive care hospitalization	1.001 (0.962–1.042)	0.953	0.003	0.953
Duration of intensive care before isolation	0.997 (0.973–1.022)	0.822	0.051	0.822
Type of infection (ref: urinary tract infection)			0.913	0.822
Ventilator-associated pneumonia	1.316 (0.572–3.028)	0.519		
Bloodstream and catheter-related bloodstream infection	1.474 (0.591–3.678)	0.406		
Wound infection	0.993 (0.256–3.846)	0.991		
Ventilator use (ref: none)	0.667 (0.361–1.230)	0.195	1.705	0.192
Central venous catheterization (ref: yes)	1.165 (0.671–2.022)	0.587	0.295	0.587
Urinary catheter (ref: yes)	0.917 (0.414–2.033)	0.832	0.045	0.832
Percutaneous endoscopic gastrostomy (ref: none)	0.543 (0.256–1.153)	0.112	2.608	0.106
Isolated Gram-negative bacteria (ref: *Pseudomonas aeruginosa*)			3.106	0.212
*Acinetobacter baumanii*	0.527 (0.214–1.302)	0.165		
*Klebsiella pnemonia*	0.811 (0.332–1.980)	0.645		
Antibiotics administered in treatment (ref: carbapenem)			1.767	0.413
Beta-lactam/beta-lactamase inhibitor	1.296 (0.652–2.577)	0.459		
Combination therapy	0.839 (0.433–1.625)	0.603		
WBC	1.000 (0.999–1.001)	0.792	0.070	0.792
Leukocyte	1.000 (0.999–1.001)	0.594	0.284	0.594
Platelets	0.998 (0.997–0.999)	0.001	12.074	0.001
CRP	1.003 (1.001–1.005)	0.049	3.891	0.049
Use of steroids in the last 30 days (ref: none)	1.591 (0.928–2.729)	0.091	2.899	0.089
Antibiotic history (ref: combination)			9.111	0.105
Carbapenem	2.459 (0.993–6.085)	0.052		
Quinolone	1.283 (0.442–3.727)	0.647		
Beta-lactam/beta-lactamase inhibitor	0.947 (0.459–1.953)	0.882		
Cephalosporin	1.094 (0.377–3.175)	0.869		
No history of antibiotics	2.793 (0.924–6.936)	0.057		
Diabetes (ref: none)	0.75 (0.401–1.402)	0.367	0.821	0.365
Surgical history in the last 30 days (ref: none)	0.552 (0.306–1.095)	0.058	4.027	0.051

Abbreviations: Exp (*β*): odds (risk) ratio; CI: confidence interval; *χ*^2^: chi-square test; *p* < 0.05: statistically significant.

**Table 3:** Distribution of isolated Gram-negative bacteria by culture type.

Culture	Isolated Gram-Negative Bacteria			*p*
	*Pseudomonas aeruginosa*	*Acinetobacter baumanii*	*Klebsiella pnemonia*	
Urine culture	1 (13)	5 (11)	11 (27)	
Tracheal aspirate culture	3 (38)	28 (61)	16 (39)	0.193
Bloodstream and catheter culture	2 (25)	10 (22)	11 (27)	
Wound site culture	2 (25)	3 (7)	3 (7)	

Abbreviations: number (*n*); percentage (*%*) values; statistically significant (*p* < 0.05).

## Data Availability

The data are contained within the article. Further inquiries can be directed to the corresponding author.

## Abbreviations

Carbapenem-resistant Gram-negative bacteria: CR-GNB; intensive care units: ICUs; C-reactive protein: CRP; *Pseudomonas aeruginosa: P. aeruginosa*; *Acinetobacter baumannii: A. baumannii*; *Klebsiella pneumoniae: K. pneumoniae*; *Escherichia coli: E. coli*; white blood cell: WBC; multidrug-resistant Gram-negative bacteria: MDR-GNB; extended-spectrum β-lactamase-producing Enterobacteriaceae: ESBL-PE; ventilator-associated pneumonia: VAP; urinary tract infections: UTIs; wound site infections: WSIs; electronic health record system: EHRS; multidrug-resistant: MDR; frequency: *n*; percentage: %; mean: X standard deviation: SD; median: M; minimum: min; maximum: max.
